# The Anticancer Effects and Therapeutic Potential of Kaempferol in Triple-Negative Breast Cancer

**DOI:** 10.3390/nu16152392

**Published:** 2024-07-23

**Authors:** Sukhmandeep Kaur, Patricia Mendonca, Karam F. A. Soliman

**Affiliations:** 1 Division of Pharmaceutical Sciences, College of Pharmacy and Pharmaceutical Sciences, Institute of Public Health, Florida A&M University, Tallahassee, FL 32307, USA; sukhmandeep1.kaur@famu.edu; 2 Department of Biology, College of Science and Technology, Florida A&M University, Tallahassee, FL 32307, USA

**Keywords:** kaempferol, flavonoids, breast cancer, TNBC, apoptosis, metastasis inhibition, chemoresistance, oxidative stress

## Abstract

Breast cancer is the second-leading cause of cancer death among women in the United States. Triple-negative breast cancer (TNBC), a subtype of breast cancer, is an aggressive phenotype that lacks estrogen (ER), progesterone (PR), and human epidermal growth (HER-2) receptors, which is challenging to treat with standardized hormonal therapy. Kaempferol is a natural flavonoid with antioxidant, anti-inflammatory, neuroprotective, and anticancer effects. Besides anti-tumorigenic, antiproliferative, and apoptotic effects, kaempferol protects non-cancerous cells. Kaempferol showed anti-breast cancer effects by inducing DNA damage and increasing caspase 3, caspase 9, and pAMT expression, modifying ROS production by Nrf2 modulation, inducing apoptosis by increasing cleaved PARP and Bax and downregulating Bcl-2 expression, inducing cell cycle arrest at the G2/M phase; inhibiting immune evasion by modulating the JAK-STAT3 pathway; and inhibiting the angiogenic and metastatic potential of tumors by downregulating MMP-3 and MMP-9 levels. Kaempferol holds promise for boosting the efficacy of anticancer agents, complementing their effects, or reversing developed chemoresistance. Exploring novel TNBC molecular targets with kaempferol could elucidate its mechanisms and identify strategies to overcome limitations for clinical application. This review summarizes the latest research on kaempferol’s potential as an anti-TNBC agent, highlighting promising but underexplored molecular pathways and delivery challenges that warrant further investigation to achieve successful clinical translation.

## 1. Introduction

Breast cancer is one of the most common cancers affecting women around the globe. It is a highly heterogeneous disease with phenotypically and genetically diverse cell groups, making it challenging to treat [1,2]. There are more than 4 million cases in the United States as of January 2022, and about 13% of women in the United States are likely to have breast cancer in their lifetime [3,4]. The molecular subtypes of breast cancer are further classified based on the expression of specific genes and proteins within the tumor cells [5]. The key risk factors for the occurrence of this disease remain gender, ethnicity, genetic makeup, lifestyle, geographical factors, and radiation exposure. There are a range of treatment strategies available to treat breast cancer, including surgical resection, chemotherapeutic agents, radiation therapy, immunotherapy, and natural compounds [6]. However, with the constant evolution and development of more resistant molecular subtypes of breast cancer, typically triple-negative breast cancer (TNBC), it has been challenging for healthcare professionals to have positive treatment outcomes [7,8]. TNBC accounts for about 10 to 15% of all diagnosed breast cancer cases. TNBC patients are typically diagnosed in the later stages of the disease due to their aggressive nature. Even with initial responsiveness towards the treatment, TNBC cases show poor prognosis, a higher rate of recurrence, and shorter disease-free survival, especially in younger women diagnosed with TNBC [9]. The conventional therapies used in the treatment of such cancer types usually fail to eradicate the cancer tumors and, hence, achieve the desired outcome, causing the cells in the tumors to persist and ultimately leading to relapse [10].

The treatment of such aggressive and resistant breast cancer types has advanced with the use of numerous multi-modality treatment strategies, making the use of combinatorial therapies or adjuvant therapies an option to fight against the resistant subtypes of cancer and, more specifically, breast cancer. However, the uncertainties associated with these treatments concerning efficacy, adverse effects, resistance, and disease recurrence remain the biggest concern. These undesirable and unavoidable treatment-related outcomes significantly lower the quality of life in the short and long term. Therefore, developing new therapies and therapeutic strategies is one of the top priorities of the healthcare sector globally [6].

In this never-ending fight against chronic diseases like cancer, natural compounds have paved their way with a vast potential to be part of the standard treatments. Natural compounds derived from multiple sources have been reported to benefit the human body for thousands of years. Still, because of the lack of reported literature and clinical evidence, they were always sidelined in the modern world of healthcare. However, in the past few decades, with the availability of resources and published literature, natural compounds have gained enormous attention for their ability to target chronic diseases like cancer uniquely; unlike synthetic drugs, which usually act on one or limited mechanisms, they can simultaneously affect multiple pathways involved in cancer development and progression [11,12]. In an exploratory concept against resistance in cancer, combinatorial therapy is also a favorable way to overcome standard therapies’ limitations. Natural compounds have been reported to target different pathways in cancer when combined with standard anticancer agents, providing synergistic effects, enhancing overall efficacy, and overcoming resistance [13].

Kaempferol, a natural flavonoid found commonly in various food items, namely tomato, tea, and broccoli, has been shown to have a variety of protective effects, including antioxidant, anti-inflammatory, cardioprotective, neuroprotective, anti-diabetic, and anticancer effects [14]. In the last few decades, kaempferol has become an important natural compound with potent anticancer activity, specifically against breast cancer. Kaempferol exhibits most of its anticancer activity by modulating multiple pathways, such as inducing apoptosis, inhibiting cell growth, and inhibiting cancer cell migration and invasion [15]. Besides its anticancer potential, kaempferol has also been studied for its synergistic potential when combined with other flavonoids or standard chemotherapeutic agents. It also protects against the adverse reactions caused by potential chemotherapeutic agents in various cancer types [16,17,18,19]. The potential of kaempferol as an anti-breast cancer agent has come a long way with an understanding of its action by modulating different molecular pathways. Still, its clinical translation as a therapy needs more robust validation to be considered part of standard adjuvant therapy [20]. 

In this review, we focus on the recently recognized work performed using kaempferol as an anticancer and chemo-preventive in human breast cancer using various in vitro and in vivo models, as well as the clinical studies that have used kaempferol as a part of their therapy. This review also aims to present the potential of kaempferol to target epigenetic modifications that render breast cancer resistant. We will further address the challenges related to its poor solubility, stability, and bioavailability and, subsequently, the efforts to address them.

## 2. Breast Cancer 

The average risk of an American woman being diagnosed with breast cancer once in her lifetime is about 13 percent, and the incidence of new cases has been increasing every year by 0.6% recently. According to the American Cancer Society’s statistics, there is an estimate of approximately 320,000 new cases of breast cancer to be diagnosed in the year 2024. Although the breast cancer-related death rate has declined by about 42% from 1989 to 2021, there are estimated to be about 42,000 deaths in women due to breast cancer in 2024 [21]. Concerning the racial and ethnic differences in the occurrence and progression of the disease, African American women are more likely to die from breast cancer at any age than Caucasians or women of any other ethnicity. The median age of African American women diagnosed with breast cancer is about 60 years, while it is 64 years for an average Caucasian woman. However, considering all these factors, about 4 million cancer survivors are living in the United States as of 2024 [4,21].

### 2.1. Breast Cancer Types

The most common classification of breast cancer divides it immunohistochemically into four subtypes: estrogen receptor (ER)-positive (+), ER-negative (−), human epidermal growth receptor (HER)+, and HER−, based on the hormonal subtype and their responsiveness towards the hormonal therapy. Another molecular subtype of breast cancer that does not express any of the aforementioned molecular receptors is TNBC [22]. TNBC has been further classified based on the genetic expression profiling of the tumor cells. Lehmann et al. (2011) explored the gene profiling of about 587 TNBC cases and performed gene cluster analysis of their genetic expressions. There are six different TNBC types with unique genetic expressions and ontologies. In this classification, there are two basal-like (BL) types, BL1 and BL2, immunomodulatory (IM) type, mesenchymal (M) type, mesenchymal stem cell-like (MSL), and luminal androgen receptor (LAR) type [23]. The authors further divided PAM50 subtyping into these six TNBC subtypes and reported their intrinsic subdivision and composition. They noted that except for MSL and LAR, all other types of TNBC are mostly composed of BL1 (99%), BL2 (95%), M (97%), and IM (84%). Whereas MSL is composed of BL (50%), normal-like (28%), and luminal B (14%), and LAR consists of HER2 (74%), and luminal B (14%). The subsequent evaluation of the subtypes for their differential prognosis revealed BL2 and M as the subtypes with the worst prognosis. LAR has the most prolonged progression and higher distant metastasis-free survival, with the maximum overall survival rate [24]. The absence of any responsive molecular targets and receptors makes TNBC relatively unresponsive to the targeted hormonal therapy and, hence, challenging to treat. It also contributes to the heterogeneous presentation of the disease. Burstein et al., 2015 also classified TNBC based on quantitative DNA expression and further classified it into four subtypes based on potential targets. Based on these targets, the classification is the LAR subtype, MSL subtype, basal-like immune-activated (BLIA) subtype (involved in signal transduction and transcription of STAT), and basal-like immunosuppressed subtype (BLIS) expressing the immunosuppressive molecule V-set domain containing T-cell activator inhibitor 1 (VTCN1) [25]. Similarly, TNBC was classified by different researchers, and each classification subgroup is characterized by unique gene expressions linked to the immune system. For example, Liu et al. used the combination of mRNA and long non-coding RNA (lncRNA) expression profiles to create the Fudan University classification (FUSCC) system, and this classification has four subtypes, namely: IM type, LAR type, M type, and BLIS type [26]. 

### 2.2. Treatment Approaches for TNBC

TNBC, being the aggressive and invasive breast cancer subtype with higher rates of recurrence and a poor prognosis, remains with adjuvant chemotherapy as the only treatment option. The standard treatment usually involves surgical resection, radiation therapy, and chemotherapy but is associated with some severe adverse effects. Apart from that, TNBC has been reported to have a higher tendency to resist the standard chemotherapeutic agents, leaving only limited therapeutic benefits from the available treatments. Also, these standard chemotherapeutic agents target only one target, such as a specific pathway, a protein, or some nucleic acids. Despite the availability of these drugs targeting various mechanisms, aggressive cancers like TNBC can still evolve and become resistant to them, which highlights the need for agents offering a more sustainable therapeutic approach [20]. 

### 2.3. Natural Compounds Used in Breast Cancer Therapy

Natural compounds have been reported and have gained immense attention in the last two decades due to their ability to target heterogeneous families of cancer cell targets and signaling pathways and simultaneously have minimal adverse effects. They are also reported to enhance the anticancer effects and lower the adverse effects of chemotherapy [27]. Many different classes of natural compounds have been evaluated for their anticancer potential. Natural products hold immense potential due to their unique mechanisms of action, but their inconsistent quality and difficulty in modification can present obstacles to treatments. The inherent structures of natural compounds do not obey the Lipinski rule of five and have a high molecular weight. Also, the notable increase in the average molecular weight of approved oral drugs over the past 20 years aligns with the growing interest in exploring natural compounds for their potential antitumor activity [28]. 

Nearly 50% of the antitumor drugs originate from natural compounds such as taxanes, vinblastine, vincristine, and podophyllotoxin analogs [29]. Also, beyond their anticancer properties, natural compounds can be used for chemoprevention since they can suppress cell growth, regulate cell division, and disrupt critical tumorigenic pathways like PI3K, MMP, MAPK/ERK, TLR, and AKT. They can activate DNA repair pathways (p21, p27, p53) and trigger the production of protective proteins (Bax, Bak, and Bid), ultimately leading to the synthesis of protective enzymes (caspases) and boosted antioxidant activity (GST, GSH, and GPx), shielding cells from damage. The chemo-preventive action of natural compounds usually comes from their potential to induce immunosurveillance (which eradicates the transforming cells), DNA repair mechanisms, apoptosis, antioxidant activity, inhibition of cell proliferation, tumor progression, and angiogenesis [29]. Natural compounds have also been found to overcome the chemoresistance developed towards standard chemotherapeutic agents by chemo-sensitizing the cells to augment the effect of chemotherapeutic agents. Natural compounds combined with chemotherapeutic agents have been shown to chemo-sensitize resistant cells and synergize the overall effect [20]. 

## 3. Kaempferol 

**Figure 1:**
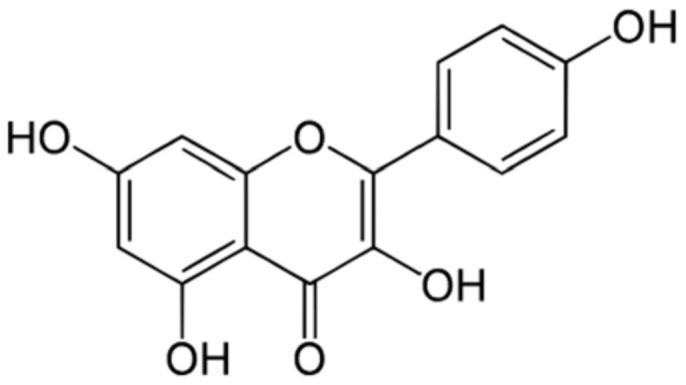
The chemical structure of kaempferol.

Flavonoids are a diverse polyphenolic group of compounds found widely distributed in various natural substances that have recently gained attention for their potential health and dietary benefits [30]. Flavonoids have a low molecular weight and are formed as a product of the shikimic acid pathway, a metabolic pathway in plants’ plastids. Flavonoids are based on carbon rings and the -OH group present in the structure and are divided into subgroups: flavanols, flavones, chalcones, flavanones, flavanonols, and isoflavones. All flavonoids from all the subclasses have a 15-carbon benzopyranone ring forming a C6-C3-C6 flavan nucleus [31,32,33] (Figure 1).

**Figure 2:**
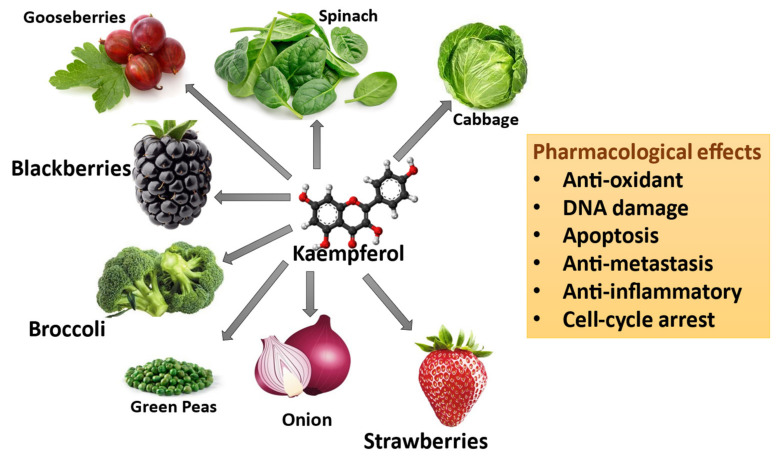
Sources and pharmacological effects of kaempferol.

Kaempferol is one of the most common and widely studied flavonoids. It belongs to the flavanol subclass and is structurally characterized by a flavone backbone with 3 4′ 5 7-tetrahydroxy groups [34]. Kaempferol was initially extracted from the rhizomes of the plant *Kaempferia galanga*. The pure compound can also be found in various plant types within the Kingdom Plantae, including Pteridophyta, Coniferophyta, and Angiosperms, while the kaempferol glycosides can be found in multiple families. It is commonly found in widely consumed vegetables like broccoli, cabbage, onion, green peas, and spinach and in berries like strawberries, gooseberries, and blackberries (Figure 2). The most consumed green leafy vegetable, spinach, contains around 55 mg of kaempferol per 100 g, and cabbage contains about 47 mg per 100 g of the vegetable. In comparison, onion has about 4.5 mg per 100 g [35]. Among spices, capers have the highest quantity, about 104.29 mg per 100 mg, compared to cloves and cumin, which have about 23.8 mg and 38.6 mg per 100 g, respectively [35].

### 3.1. Kaempferol Absorption and Metabolism

After being absorbed into the body, kaempferol undergoes extensive metabolism in the liver and converts to either methyl or sulfate salts as glucuronide-conjugated metabolites, which then circulate in the blood. Kaempferol is also metabolized by the intestinal microbiota into 4- 4-phloroglucinol, hydroxyphenyl acetic acid, and 4-methylphenol compounds, and then enters the systemic circulation. It has also been reported that kaempferol acts as a precursor of the CoQ ring in renal cells, thereby increasing concentration in the kidney cells and contributing to the production of ubiquinone. It is proposed to be responsible for its antioxidant properties. Although kaempferol has also been seen being excreted unchanged in the urine, the other metabolic products of kaempferol, both formed as free aglycone products or as O- or C-glycosides, are reported to be excreted in urine and feces [31,35,36]. 

### 3.2. Kaempferol Pharmacokinetics

Kaempferol has poor water solubility and has been studied extensively for its pharmacokinetic properties both in vitro and in vivo. It is mainly absorbed through the small intestine and has passive diffusion because of its lipophilic nature. Because of the multiple sugars in its structure, the membrane-bound beta-glucosidase in enterocytes removes the terminal saccharides and exposes the glucose, which is absorbed [32]. Kaempferol is metabolized in the liver and undergoes phase I (oxidation) and phase II (glucuronidation, sulfation) metabolism. The flora in the large intestine metabolizes the kaempferol glycosides into aglycones and then to some phenolic compounds, which are absorbed in the systemic circulation, distributed in various tissues, and excreted in feces and urine. It has been reported that kaempferol offers a vast therapeutic advantage, even with limited oral bioavailability [31].

### 3.3. Pharmacological and Toxicological Properties of Kaempferol

Kaempferol has been proven to have a variety of health benefits ranging from cardioprotective, neuroprotective, chemo-preventive, anticancer effects, and protective effects against various metabolic disorders, even when taken as a dietary substitute or as a part of adjuvant therapy [37,38]. It has been traditionally used as a dietary supplement for its antioxidant and anti-inflammatory properties [39]. 

Kaempferol has been explored for many years as a treatment for its anti-inflammatory properties. Chronic inflammation, which is the culprit of multiple diseases, namely cardiovascular diseases, metabolic and autoimmune disorders, and even cancer, can be targeted by using natural products like kaempferol [40,41]. Kaempferol specifically targets oxidative stress and modulates pro-inflammatory enzyme activities, genetic expression of the genes involved in inflammation, inhibition of transcription factors involved in inflammatory pathways, and other mechanisms involved in inflammation [40]. 

Besides its active moiety, kaempferol can modulate physiological effects with its other bioactive metabolites. With its interaction with free radical generation in an oxidative stress environment, kaempferol tends to have pro-oxidant activity and produce genotoxic effects [40]. Various antioxidants and pro-oxidant enzymes regulate these pro-oxidant mechanisms. Kaempferol has also been reported to have an impact by reducing metal ion concentrations with its antioxidant activities and affecting iron concentrations and folic acid bioavailability. Despite the multiple studies addressing kaempferol’s mutagenic and carcinogenic potential, there is limited in vivo evidence corroborating the effects [37,40,42]. 

Kaempferol is relatively non-toxic to healthy human cells, unlike the standard chemotherapeutic agents. It has been evaluated for its activity in healthy human breast epithelial cells (MCF-10A cells) and other class flavonoids. As indicated in a cytotoxicity study by Mohd. Afzal et al., 2013, kaempferol in combination with fisetin was safe for the cells and showed no significant toxicity [16]. They also analyzed the activity of kaempferol alone towards MCF-10A cells and found it to be safe with no cytotoxic potential towards healthy breast epithelium cells [16]. Interestingly, clinical studies performed on healthy individuals to analyze the nutritional benefits of consuming food items rich in kaempferol also signify the potential benefits of kaempferol against chronic inflammation, which has emerged as a culprit for multiple chronic diseases like cancer [32].

## 4. Anticancer Effects of Kaempferol in Human Cancers

The consumption of flavonoids like kaempferol in the form of fruits and vegetables is linked to a decreased incidence of various human cancers. Kaempferol and other flavonoids exert anticancer effects by modulating multiple physiological pathways. As part of various traditional medicinal systems and dietary habits across different communities, kaempferol emerged to have anti-tumorigenic and chemo-preventive effects in the 1970s and 1980s [43,44]. The subsequent development in understanding the mechanisms underlying the development and recurrence of cancers allowed the researchers to explore the potential benefits of using kaempferol against various human cancer types [45]. 

Initially, scavenging oxidative stress and modulating inflammatory pathways were primarily targeted for the anticancer activity of kaempferol [46,47,48]. Its antioxidant and anti-inflammatory properties were observed in hydrazine- and H_2_O_2_-induced colorectal and hepatoma cancer cell models [48,49]. Besides exerting anti-tumorigenic, antiproliferative, and apoptotic effects against cancer cells, kaempferol also showed protective activity towards healthy, non-cancerous cells. Early in evaluating kaempferol as an anticancer agent, Nguyen et al., 2003, showed inhibition in the growth of A549 lung cancer cells with kaempferol treatment in a dose-dependent manner. They also showed that kaempferol-induced apoptosis was validated by increased and decreased pro-apoptotic and anti-apoptotic protein expression, respectively. Further, they confirmed the activation of MEK-MAPK-induced apoptosis in A549 cells [50]. Subsequently, Zhang et al., 2008, reported similar results with kaempferol obtained from ginkgo biloba extract in pancreatic cancer cells [51,52]. Wang et al., 2021, in their study, showed ROS-dependent induction of apoptosis in in vitro and in vivo models of pancreatic cancer. They mainly showed that this induction of anticancer activity with ROS generation was via tissue transglutaminase (TGM2)-mediated Akt/mTOR signaling [53]. Subsequently, the potential anti-tumorigenic effects of kaempferol were further seen in different in vitro and in vivo models of human cancer, targeting other more cancer-specific molecular targets. Lee et al., 2016, in their in vitro study using kaempferol as a treatment against human pancreatic cancer cell lines Panc-1, Miapaca-2, and SNU-213, showed significant cytotoxic action in a dose-dependent manner. They also showed that the anti-migratory action of kaempferol was mediated by the inhibition of EGFR-related Src and ERK1/2/AKT pathways [54]. Similarly, several other studies showed the dose-dependent inhibition of cell viability and kaempferol-induced apoptosis via the P13/AKT hTERT pathway in various human cancer types [54,55,56]. Yao et al., 2016 showed that kaempferol induced G0/G1 cell cycle arrest. Their mechanistic studies showed a significant downregulation of EGFR signaling in an in vitro model of esophagus squamous cell carcinoma (ESCC). They also demonstrated decreased aerobic glycolysis, markedly seen in the tumor environment, with kaempferol treatment in the ESCC in vitro model. The in vivo xenograft model of ESCC further confirmed their observations [57]. Similarly, another study showed kaempferol-inducing apoptosis effects and cell cycle arrest in in vitro models of human renal carcinoma cells. They showed the EGFR/p38 signaling pathway and upregulation and downregulation of p21 and cyclin B1 expressions, respectively. Their cell growth inhibition and apoptosis were linked to the activation of PARP cleavage [58]. In an in vitro model of osteosarcoma cells (US-2 OS), apart from showing specific cytotoxic action against the OS cells and sparing the human fetal osteoblast progenitor cells (hFOB cells), the authors reported the induction of apoptosis with a significant increase in cytoplasmic Ca^2+^ and decrease in mitochondria membrane potential (Δψm) levels in US-2 OS cells, as demonstrated by the DiOC6-based flow-cytometric assay. They also showed the induction of various endoplasmic reticulum stress-related proteins and apoptotic proteins, including GRP78, GRP94, GADD153, ATF-6α, ATF-6β, caspase-4, caspase-12, calpain 1, caspase 3, and caspase 6 activity [57]. Yet, another group reported the induction of apoptosis and G2/M cell cycle arrest in human ovarian carcinoma A2780/CP70 cells with kaempferol treatment. They particularly observed that kaempferol-induced G2/M cell cycle arrest in their in vitro model of ovarian carcinoma was mediated by the Chk2/p21/Cdc2 and Chk2/Cdc25C/Cdc2 pathways [59]. Kaempferol could also induce anti-angiogenic potential, which is another major clinical challenge faced in cancer therapy. In 2009, a study by Luo and colleagues focused on analyzing the anti-angiogenic effect of kaempferol against ovarian cancer and reported that although kaempferol could not affect the viability of ovarian cancer cells significantly, it could inhibit angiogenesis and angiogenic proteins. The marked decrease in the mRNA and protein levels of Vascular Endothelial Growth Factor (VEGF), a potent anti-angiogenic factor caused by kaempferol, was also able to downregulate the expression of HIF-α (a regulator of VEGF) [60]. In a study by Chin et al., 2018, kaempferol decreased VEGF-stimulated human umbilical vein endothelial cell (HUVECs) viability, invasion, migration, and tube formation, and the angiogenic inhibition of kaempferol was related to the regulation of VEGF/VEGFR-2 and PI3K/AKT, MEK, and ERK pathways in VEGF-stimulated HUVECs [61]. Subsequently, kaempferol has also been shown to inhibit the migration and invasion of different human cancer cell types. Ju et al., 2021, in their study on human hepatocellular carcinoma cells (HCC; Huh-7 and SK-Hep-1 cells), reported that the treatment of kaempferol significantly reduced and suppressed the viability, migration, and invasion of the HCCs, and that this action of kaempferol was backed by decreased activity of MMP9, Cathepsin C, Cathepsin D, and phosphorylated AKT (pAKT) [62]. Amble literature indicates kaempferol’s anticancer effect in many cancer cell types, making it an ideal candidate to be developed for its clinical use. 

### 4.1. Kaempferol in Combinatorial Drug Therapy

Kaempferol has also been explored for its synergistic activity with other therapeutic agents, especially when given with chemotherapeutic agents. It has specifically been reported to increase chemo-sensitization in resistant cancer cells. The idea of combining kaempferol with standard chemotherapeutic agents came when some of the structurally related flavonoids were studied when combined with standard chemotherapeutic drugs in a study by Cipak et al., 2003 [63]. They reported that flavonoids differentially modulate the anticancer activity of doxorubicin by decreasing its cytotoxic activity and by shifting the cell cycle arrest from the G2/M phase to the S phase of the cell cycle [63]. It was then reported to downregulate the expression of cMyc, a regulator of cell proliferation and apoptosis but involved in drug resistance, in ovarian cancer cells [64]. Similarly, kaempferol, when used in combination with 5-fluorouracil, docetaxel, and cisplatin, showed additive effects towards pancreatic cancer, prostate cancer cells, and head and neck squamous cell carcinoma [52,65]. Additionally, the potential of kaempferol, used in combination with other natural drugs to target chronic diseases like cancer, was also explored, as it may affect multiple pathways involved in the disease process [66]. Kaempferol, in combination with another dietary flavonol, fisetin, showed synergistic cytotoxic activity and induction of apoptosis at lesser doses than when used individually [16].

### 4.2. Kaempferol Reversal of the Chemoresistance of Chemotherapeutic Agents

The standard anticancer therapies, including chemotherapy and radiotherapy, offer generous clinical outcomes, increasing overall survival rates in patients, but are associated with reduced efficacy and the development of resistance over time. The induction of drug-resistance proteins and epigenetic mechanisms are involved in the development of chemoresistance [14]. For many years, kaempferol has been evaluated by multiple research groups for its potential to reverse chemoresistance and synergize the anticancer activity of agents [67]. Using various mechanisms, including the inhibition of ROS generation and oxidative stress, kaempferol can reverse the chemoresistance of standard chemotherapeutic agents. In a study by Ichrak Riahi-Chebbi et al., 2019, apart from inhibiting and reversing the resistance, kaempferol also showed synergism with increased anticancer activity when given in combination with 5-fluorouracil (5-FU) in resistant human LS174 colon cancer cells [68]. Their findings demonstrated that kaempferol, alone or combined with 5-FU, induced apoptosis, cell cycle arrest, and suppressed reactive oxygen species (ROS) production. Additionally, kaempferol modulated the expression of key signaling pathways, including JAK/STAT3, MAPK, PI3K/AKT, and NF-κB, suggesting its potential role in reversing drug resistance [68]. Another study did so by suppressing glucose uptake and lactate production in human colorectal cancer cells. Mechanistically, it induced a pronounced upregulation of microRNA-326 (miR-326) and inhibited the expression of pyruvate kinase M2 isoform (PKM2) [69]. Similarly, kaempferol reversed the oxaliplatin resistance in human colon cancer cells by inhibiting the expression of A Jun and Fos heterodimers (AP-1), which are involved in growth factor-mediated cell cycle progression and cell proliferation [70]. Interestingly, Sourav Kumar Nandi et al., 2023, in their study, used the combination of kaempferol and verapamil to impede the chemoevasion in their ex vivo model of breast cancer, as demonstrated by the downregulation of resistance-related markers. In particular, the authors demonstrated that the combination of kaempferol and verapamil induced a significant overproduction of ROS and downregulation of chemoresistance and tumor acidosis markers. The treatment with kaempferol resulted in decreased expression of ATP1B1 (a regulator of cellular membrane potential) and disrupted lysosomal function [71]. Kaempferol’s pleiotropic effects on reversing chemoresistance in human cancers, mediated through modulation of multiple molecular pathways, warrant further investigation as a potential therapeutic strategy to overcome resistance in TNBC.

## 5. Kaempferol Anticancer Mechanisms of Action in Breast Cancer 

Kaempferol has been used to target multiple molecular pathways in human breast cancer. Eating foods rich in kaempferol and kaempferol-like flavanols is associated with a reduced risk of breast cancer [72,73]. It was in the 1990s that the varied effects of kaempferol and other flavonoids on human breast cancer were initially evaluated, followed by a determination and analysis of their potential. Kaempferol has been reported to target multiple pathways to produce the desired anticancer activity. Interestingly, being the most commonly used dietary phytoestrogen, kaempferol also possesses the ability to modulate estrogen-mediated activity, which acts as one of the principal mediators of hormone-dependent breast cancers. Similarly, kaempferol has been shown to have promising anticancer effects against TNBC by modulating more than one molecular pathway [74,75]. 

### 5.1. Effect of Kaempferol on DNA Synthesis Inhibition

As the development of breast cancer has also been historically linked to lifetime exposure to both endogenous and exogenous estrogen, kaempferol has been widely studied for its anticancer activity mediated by estrogen-dependent pathways and in cancer types independent of estrogen [76]. Wang et al., 1997, analyzed the effect of kaempferol on DNA synthesis in human estrogen-dependent (ER+) (MCF-7) and estrogen-independent (ER−) (MDA-MB-231) breast cancer cells. They showed the dose-dependent effects of kaempferol on DNA synthesis in breast cancer cells, in which kaempferol promoted DNA synthesis at very low concentrations. At the same time, it significantly inhibited DNA synthesis at a concentration of around 50 µM compared to the control cells [77]. Similarly, Zava et al., 1997 and 2023, replicated the action of kaempferol alongside other flavonoids in other ER+ (MCF-7 and T47D) and ER− (MDA-MB-468) breast cancer cells and reported that kaempferol shows estrogen agonistic activity. It has concentration-dependent DNA inhibition and cell growth inhibition [78].

Interestingly, kaempferol displayed notable estrogenic effects, enhancing the transcriptional activity of the estrogen receptor among other flavonoids when evaluated for estrogenic activity using an engineered yeast strain with human estrogen receptor integration. Although its potency was 4000–4,000,000 times lower than natural estrogen, its activity was comparable to certain established isoflavonoid estrogens, and its estrogenic potential was validated in estrogen-dependent MCF7 breast cancer cells [78]. Interestingly, kaempferol exhibited both estrogenic and antiestrogenic properties, showing a two-phase response on the estrogen receptor. In a study by Min oh et al., 2006, the authors reported that at lower concentrations (as low as 10–12 M), it acts as an estrogenic agent through the estrogen receptor-mediated pathway, and at higher concentrations (as high as 10^−4^ M), kaempferol exerts strong antiproliferative effects, independent of estrogen receptor activity. This also suggests that kaempferol can inhibit cancer cell proliferation via an estrogen-dependent pathway, indicating its potential to prevent malignant transformations driven by estrogen exposure and, hence, its ability to maintain a balanced estrogenic activity [74]. Similarly, Hung et al., 2004, showed with MCF-7 cells that kaempferol decreased cell viability in a dose- and time-dependent manner. It also notably decreased ER-alpha mRNA and protein levels, reducing estrogen-responsive gene expression. At the same time, they showed reduced expression of the progesterone receptor, cyclin D1, and insulin receptor substrate and that kaempferol induced ER-alpha protein aggregation and degradation through a distinct pathway compared to that of estradiol [79]. 

Interestingly, kaempferol has also been reported to have anticancer and antiproliferative properties in ER receptor-mediated triclosan-induced cell growth in MCF-7 cells. Kim et al., 2016 demonstrated the antiproliferative activity of kaempferol in triclosan-induced cell viability of MCF-7 cells and in a xenograft mouse model by using various combinations of an ER antagonist (ICI 182,780), an ERα agonist (Propyl pyrazole triol, PPT), and an ERβ agonist (diaryl propionitrile, DPN) [80]. They further analyzed the effect of kaempferol on triclosan or estradiol-induced activation of the IGF-1 signaling pathway and observed that the treatment of kaempferol in combination with triclosan, and estradiol significantly downregulated the expression of the proteins pAkt, pMEK1/2, pIRS-1, and pERK1/2 [80]. In another study, kaempferol showed inhibition of cancer cell migration in the triclosan and estradiol-induced cell growth, demonstrated using a wound healing assay and suppressed trans-well, and inhibited cell invasion when given in combination with the triclosan and estradiol in a cell invasion assay. This suppression demonstrates the inhibition of metastatic and epithelial–mesenchymal transition (EMT), which was further confirmed by the downregulation of the increased expression of EMT-related markers: N-cadherin, Snail, and Slug, and metastasis-related proteins: Cathepsin B, Cathepsin D, MMP-2, and MMP-9 in triclosan and estradiol-induced MCF-7 cells [81]. 

### 5.2. Effect of Kaempferol on ROS Production

Various anticancer treatments have been studied for their anti-oxidative properties to neutralize free radicals and the oxidative stress caused by irregular cellular function and metabolism. Mitochondrial dysfunction has emerged as one of the prominent culprits for the increased oxidative stress in the body and in and around the tumor sites, which results in cancer initiation and progression. The ROS encompasses a group of oxygen-containing molecules exhibiting high chemical reactivity. Prominent members of the ROS family include free radicals like hydroxyl and superoxide, along with hydrogen peroxide [82]. Cellular production of ROS occurs physiologically within the mitochondrial electron transport chain, peroxisomes, and phagosomes, contributing to energy generation and phagocytosis. In addition to this, the NADPH oxidase family in the plasma membrane generates ROS and participates in various intracellular signaling pathways, including those involving Ras, c-Jun N-terminal kinase (JNK), p38, mitogen-activated protein kinase (MAPK), and PI3K/AKT/mTOR. ROS also play a significant role in cell proliferation, along with processes like autophagy, apoptosis, and inflammation mediated by the NLRP3 inflammasome and the nuclear factor-κB (NF-κB) pathway [83]. The malignant transformation of the cells, the formation of tumors, and their progression and metastasis are part of a vicious cycle with oxidative stress. Tumors undergo vascularization via angiogenesis to meet the growing demands of oxygen and nutrients for sustained proliferation. ROS, mainly hydrogen peroxide (H_2_O_2_), plays a crucial role in this process. ROS promote vascularization by enhancing the activation of vascular endothelial growth factor (VEGF) and increasing the production of matrix proteins. Endothelial cells primarily generate ROS through NADPH oxidase, further contributing to the upregulation of hypoxia-inducible factor 1α (HIF-1α) expression [84].

Additionally, the generation of estrogen-dependent oxidative metabolism has also been reported to be involved in generating ROS implicated in the carcinogenic transformation and growth of cancer cells, which suggests the existence of additional mechanisms, independent of ER status, that mediate estrogen-induced cell signaling leading to malignant transformation and growth of mammary epithelial cells. Estrogen-induced mitochondrial ROS is a mechanism involved in mammary carcinogenesis [85]. In a study by Quentin Felty et al., 2005, the physiologically relevant estrogen concentrations corresponding to the menstrual peak demonstrated the rapid mitochondrial stimulation and generation of ROS in MCF-7 cells [86]. Interestingly, a convergence exists between mitogenic pathways sensitive to ROS levels and those regulated by carcinogenic estrogen concentrations. The inhibitors of mitochondrial ROS production abrogate the E2-induced expression of cell cycle genes harboring nuclear respiratory factor-1 (NRF1) binding sites, such as cyclin B1, PCNA, and PRC1, and these inhibitors suppress E2-induced NRF1 expression and, consequently, delay cellular proliferation [86]. 

Over the decades of cancer research, multiple therapeutic strategies modulate ROS levels and have demonstrated promising efficacy in both in vitro and in vivo cancer models. These strategies encompass approaches that either scavenge ROS or promote their generation within cancer cells, as shown in both in vitro and in vivo models of different human cancers [84]. Kaempferol’s mechanism of action appears to be multifaceted, and multiple studies have demonstrated its ability to considerably impede the development of various inflammatory processes by suppressing ROS generation and exhibiting high anti-oxidative properties. It also inhibits the expression of pro-inflammatory cytokines IL-1β and TNF-α and disrupts the translocation of NF-κB into the nucleus, thereby hindering the production of inflammatory proteins [87]. Apart from the multiple molecular targets it acts on, the antioxidant activity of kaempferol is also attributed to the presence of hydroxyl groups within its molecular structure, particularly the one located at the C-3 position. Apart from the direct elimination of ROS, kaempferol also becomes involved in preserving endogenous antioxidant enzymes like glutathione peroxidase, superoxide dismutase, and catalase at normal physiological levels [87]. In another study by Jie Zeng et al., 2020, kaempferol inhibited breast tumor metastasis in both in vitro and in vivo models of 4T1 breast cancer cells by inhibiting the neutrophil extracellular trap. Neutrophils are the first cells to be recruited at the site of inflammation; the high neutrophil-to-lymphocyte ratio has been reported to be associated with higher metastasis and poor disease outcomes in breast cancer [88]. Moreover, kaempferol has also exhibited synergism when combined with other anticancer agents by inhibiting intracellular ROS generation as one of their mechanisms of action using various in vitro models of MDA-MB-231 cells. The combination of kaempferol and fisetin induced the activation of γ-H2AX, a histone variant, which, by causing DNA damage, leads to apoptosis in TNBC cells [16].

Interestingly, kaempferol also helps alleviate the serious adverse effects of standard chemotherapeutic agents. The standard chemotherapeutic drug doxorubicin is reported to have excessive ROS-mediated cardiotoxicity and endotheliotoxicity as its prominent adverse reactions. Wu et al., 2020, in their study, showed that kaempferol reversed the vascular doxorubicin-induced vascular toxicity by reducing oxidative stress, improving mitochondrial function by regulating the levels of 14-3-3 proteins, conserving regulatory molecules of eukaryotic cells and controlling the levels of various oxidative stress molecules [19]. Conversely, kaempferol has also been reported to induce apoptosis, with the production of ROS leading to apoptosis. In a study by Bong-Woo Kim et al., 2008, kaempferol induced apoptosis by modulating the ROS-mediated ERK/MEK1/ELK1 signaling pathway in breast cancer cells [89].

Many studies have investigated the anti-oxidative potential of kaempferol and its application as an anti-breast cancer agent. The multi-faceted mechanism of action associated with kaempferol allows it to effectively inhibit multiple ROS species, making it an ideal antioxidant agent.

### 5.3. Effect of Kaempferol on Nrf2 Activation

Nuclear factor-2 (Nrf2) is a protein regulator of cellular oxidative response in both normal and malignant cells. It protects the healthy cells from oxidative stress, thereby preventing their malignant transformation, and guards the malignant cells against radiation and chemotherapy, resulting in the development of chemoresistance. Nrf2 neutralizes ROS or repairs cellular damage caused by oxidative stress. It facilitates the rapid enzymatic detoxification and elimination of carcinogenic chemicals, but once the cancerous tumors have formed, Nrf2 becomes involved in the cancer progression and metastasis. This surprising phenomenon was later described as the “dark side” of Nrf2 [90]. In the absence of cellular stress, Nrf2 is localized within the cytoplasm. In this basal state, Nrf2 remains tethered to its negative regulatory protein, Kelch-like ECH-associated protein 1 (Keap1) [91]. On becoming exposed to oxidative stress, it undergoes nuclear translocation, where it heterodimerizes with avian musculoaponeurotic fibrosarcoma oncogene homolog (sMAF), forming a complex that subsequently binds to specific DNA sequences known as antioxidant-responsive elements (AREs), and with this interaction, Nrf2 regulates the transcription of genes involved in intracellular redox balance, metabolism, apoptosis, and autophagy. On the dark side, the chronic activation of Nrf2 leads to increased gene expression in drug metabolism, fostering resistance to chemotherapeutic agents and radiotherapy [92].

Furthermore, Nrf2 hyperactivation promotes cellular proliferation by inducing metabolic reprogramming towards anabolic pathways, modulating the pentose phosphate pathway (PPP, crucial for generating precursors for nucleic acid synthesis), and augmenting purine synthesis, which is essential for DNA replication and cell division. The dysregulation of the Nrf2-Keap1 signaling pathway has been observed in a broad spectrum of human malignancies, including breast cancer, and multiple downstream signaling pathways are involved in disease pathogenesis, including increased Nrf2 levels, decreased Keap1 levels, and blocked Nrf2 ubiquitination. Many molecules of natural origin have been used to modulate Nrf2 activity by disrupting the intermolecular disulfide bonds formed between two cysteine residues (Cys273 and Cys288) within the Keap1 protein, which weakens its ability to sequester Nrf2 in the cytoplasm, thereby facilitating Nrf2’s nuclear accumulation and subsequent activation of antioxidant response pathways [92,93]. Kaempferol has been reported to activate Nrf2 and its downstream signaling pathways in multiple human cancer cell models, including MCF-7 breast cancer cells [15,53]. It modulates the Nrf2-ARE signaling pathway, thereby activating Nrf2 expression and the antioxidant response. However, in other models of human cancer, kaempferol has been seen to downregulate the expression of Nrf2, demonstrating kaempferol’s action against the dark side of Nrf2. In a study by Foudzer et al., 2021, the authors showed that in NSCLC cells, kaempferol inhibited Nrf2 and induced ROS accumulation after 48 h of treatment, thereby making the NSCLC cells sensitive to apoptosis at physiological concentrations [94]. Nrf2 has attracted significant interest as a potential therapeutic target for several years, owing to its role in cancer progression. Notably, kaempferol exhibits a paradoxical effect on Nrf2 expression, demonstrating upregulation and inhibition in various human cancers [53,94]. This intriguing observation underscores the need for further exploration to elucidate the underlying mechanisms by which kaempferol modulates Nrf2 expression. Understanding these mechanisms is crucial for harnessing the full therapeutic potential of kaempferol in cancer treatment.

### 5.4. Effect of Kaempferol on Cell Cycle Arrest

Kaempferol causes cell growth inhibition by inducing cell cycle arrest in TNBC cells. It particularly induces cell cycle arrest at the G2/M phase of the cell cycle when treated for about 48 h in MDA-MB-468 and MDA-MB-231 cells [43,95]. It also produces the same effect on MDA-MB-453, androgen-responsive human breast carcinoma cells extensively used in TNBC research, particularly by downregulating the cyclin-dependent kinase 1 (CDK1) and the associated proteins cyclin A and cyclin B [96,97]. In another study, the chloroform extract of the *Butea monosperma* (Lam.) Taub bark, an Indian medicinal plant rich in kaempferol, showed arrest in the G1 phase of the cell cycle in MCF-7 cells in a concentration-dependent manner [98]. In Kim et al., 2016, the authors represented the efficacy of kaempferol in inducing cell cycle arrest by downregulating the expression of cyclin D1 and cyclin E and upregulating the expression of p21 in triclosan and estradiol-induced ER-mediated increased cell proliferation of MCF-7 cells and the in vivo mouse model [80].

### 5.5. Effect of Kaempferol on Apoptosis Induction

Induction of apoptosis in cancer cells is one of the principal mechanisms of kaempferol anticancer activity. Induction of apoptosis is also linked with cdc2 dephosphorylation and cyclin B1 downregulation, which alter cell cycle kinetics in breast cancer cells. Kaempferol was initially shown to induce apoptosis in MDA-MB-231 cells using the trypan blue staining method when treated in a different concentration-dependent manner, and it showed maximum effect at 48 h of incubation. The results were confirmed with kaempferol-induced chromatin condensation and formation of oligonucleotides due to nuclei fragmentation at a 50 µM concentration, which showed increased expression of cleaved PARP in kaempferol (50 µM) treated MDA-MB-231 cells at 24 h [99]. PARP is a protein that is involved in both DNA repair and apoptosis, is cleaved by caspases, and acts as an apoptotic marker. Pro-apoptotic protein Bax and anti-apoptotic protein Bcl2 also serve as indicators of programmed cell death. Kaempferol extracted from various natural sources commonly used by humans and non-human primates has also been reported to show its antitumor activity via the induction of apoptosis demonstrated by activation of multiple caspases, increased expression of cleaved PARP, phosphorylated DNA damage associated protein ATM (pATM), and pro-apoptotic protein Bax, and decreased expression of anti-apoptotic protein Bcl-2 in MCF-7 and MDA-MB-231 cells [16,95,100,101,102,103,104]. 

Kaempferol apoptotic effect has been confirmed with the formation of condensed DNA, DAPI staining, and increased expression of phosphorylated H2A histone family member X [75]. Diverse subfamilies of MAPKs involved in apoptosis have been reported to be activated and responsible for the induction of apoptosis. Along with the activation of cleaved PARP, treatment of kaempferol in MDA-MB-231 and MCF-7 cells showed activation of extracellular signal-regulated kinase (ERK), evident with increased expression of ERK and phosphorylated ERK (pERK). The study showed the simultaneous activation of the upstream kinase and a substrate of ERK, as evidenced by the increased expressions of MEK1 and pMEK1 and ELK1 and pELK1. The inactivation of ERK using a MEK1 inhibitor (PD98059) and transfecting the MCF-7 cells with a kinase-inactive ERK mutant (ERK-DN (K52R)) showed marked suppression of apoptosis, as demonstrated by the decreased expression of cleaved PARP in the kaempferol treated cells. The induction of apoptosis via the modulation of ERK and its suppression by its activation was also reported to be more profoundly evident in 3D-cultured MCF-7 cells [89]. Kaempferol also downregulates polo-like kinase-1 (PLK-1) expression in MCF-7 cells, another mammalian protein kinase and a key regulator of mitosis. It has been reported to be involved in tumor induction and tumor progression and is overexpressed in a variety of tumors, including breast cancer [105]. Interestingly, kaempferol has also been reported to cause induction of apoptosis, as demonstrated by the increased expression of Bax and decreased expression of Cathepsin D (an essential lysosomal aspartic protease involved in breast cancer metastasis) in triclosan and estradiol-treated MCF-7 cells and a xenograft mouse model [80].

Alternatively, studies have also demonstrated the potential of kaempferol to reverse the ability of aggressive breast cancer tumors to escape apoptosis and possess stemness. The TCGA database analysis revealed a correlation between the levels of p53, a tumor suppressor protein, and caspase 3, where the two levels were higher in breast tumors than in normal cells [71,106]. Whereas, in a study by Nandi et al., 2022 on a cohort of 271 female breast cancer tissues, higher levels of p53 and lower levels of caspase 3 have been reported with advanced stages (stage II/IV) of the disease, while the vice versa is associated with lower stages (stage I/II). The high expression levels of p53 and downregulated caspase 3 expression have also been reported in multiple studies to be related to more advanced metastases with nodular-involved cancer stages. In usual conditions, the activation of caspase 3 leads to the induction of apoptosis and results in cell death. However, in p53-mutated cells, the activation of caspase 3 does not initiate apoptosis, which causes chemoresistance. Mutant p53 leading to its deactivation is also associated with increased stemness and expression of MDR1 and various anti-apoptotic proteins, including ALDH1, NANOG, NF-κB, CD 44, Ki-67, and Bcl2. The former cohort of 217 breast cancer patients showed more co-expression between p53 mutant and Ki-67 than other anti-apoptotic proteins. Interestingly, the treatment of ex vivo neo-adjuvant chemotherapy-treated primary human breast cancer tissues with kaempferol showed a notable downregulation of p53, ki-67, NANOG, NF-κB, CD 44, ALDH1, Bcl2, and upregulation in the expression of caspase 3 in comparison to the treatment with carboplatin, which indicates the ability of kaempferol to reverse the acquired chemo-tolerance in advanced stage breast cancer tumors [71]. 

Subsequently, kaempferol was further shown to inhibit breast cancer cell proliferation via diversified mechanisms. For instance, Brusselmans et al., 2005, showed that kaempferol inhibited fatty acid synthase, a prominent lipogenic enzyme found overexpressed in human cancers, correlating the effect of kaempferol with reduced cell growth and increased apoptosis [99].

### 5.6. Effect of Kaempferol on Cell Invasion and Metastasis Inhibition

Concerning inhibiting migration and invasion, kaempferol has also been described as having anti-metastatic activity [107]. Astin et al., 2014, in their study, reported, for the first time, kaempferol to be a novel inhibitor of Vascular Endothelial Growth Factor Receptor (VEGFR) kinases, which are involved in increased vascular permeability and angiogenesis, and they reported kaempferol anti-lymphangiogenic activity in their zebrafish model of lymphangiogenesis [108]. Kaempferol, especially in its lower doses, has shown promising effects in TNBC compared to other non-TNBC in vitro models. In a study by Shoushan Li et al., 2017, the authors showed the suppression of migration with low-dose kaempferol treatment in an in vitro model of MDA-MB-231 and MDA-MB-453 cells in comparison to MCF-7 and SK-BR-3 cells. They mainly reported the inhibition of activation of RhoA and Rac, small GTP-binding proteins primarily involved in microfilament rearrangement and cancer cell migration, with low doses of kaempferol treatment in TNBC cells [95]. In another study, kaempferol inhibited cell adhesion, cell motility, and cell migration in various in vitro assays using MDA-MB-231 cells. Kaempferol also significantly downregulated the expression of matrix metalloproteinases-2 (MMP-2) and MMP-9, molecules involved in ECM degradation, cell invasion, and cancer metastasis, compared to the control. The activity of MMPs is regulated by either the signal transducer and activator of transcription 3 (STAT3) or activator protein-1 (Ap-1) pathways. Kaempferol-treated MDA-MB-231 showed inhibition in the nuclear translocation of cJun and cFos to increased cytoplasmic levels, and both cJun and cFos are components of Ap-1. The further downstream pathway analysis showed downregulation of MMPs via AP-1 induction mediated activation of MAPK and the protein kinase Cδ (PKC-δ) signaling pathway in kaempferol-treated cells. Li et al., 2015, also reported the inhibition of lung metastasis with kaempferol when used in doses as high as 200 mg/kg compared to decarbazine at 100 mg/kg. They also reported a notable decrease in the expression of MMP-9 in kaempferol (200 mg/kg)-treated lung tissue nodules [109]. 

### 5.7. Kaempferol Epigenetic Modulation

The complex nature of cancer due to the underlying complex mechanisms makes it difficult to target and find an ultimate cure for the disease. Various pathways and mechanisms have been explored using a variety of interventions for years to fight multiple aspects of cancer. Cancer research to date has investigated diverse strategies ranging from cure (eradication, reduced aggressiveness), prevention (risk factor targeting, pre-cancer intervention), and management (reduced side effects, preventing recurrence) post-treatment [110]. 

Apart from genetics, epigenetic factors are broader in implicating cancer development and influencing its aggressiveness. Epigenetic changes are transmissible changes in chromatin and gene expression without causing any real change in the DNA sequence. However, they can still make characteristic changes in the cellular processes and the phenotype. Typical epigenetic modifications include chromatin remodeling, histone modifications, acetylation, methylation, phosphorylation, ADP-ribosylation, ubiquitination, and non-coding RNAs (ncRNAs), which can either result in the induction or suppression of gene transcription, specifically oncogene transcription, in cancer and therefore, play a crucial role in the induction and progression of cancer [111]. 

Due to its remarkable anticancer activity, kaempferol has been evaluated in multiple studies for its potential activity in modulating epigenetic regulation. In an in silico survey by Berger et al., kaempferol was reported to have HDAC inhibitory activity in human hepatoma cells (HepG2 and Hep3B). The in silico docking reported that kaempferol fits in the binding pocket of HDAC-2, 4, 7, or 8 and binds to the Zn ion in the enzyme’s catalytic core. The authors further evaluated their results in in vitro models of human hepatoma cells [112]. Kim et al., 2020, explored kaempferol effects on gastric cancer cells, revealing its ability to induce autophagy and cell death through the IRE1-JNK-CHOP signaling pathway, potentially via epigenetic modulation involving G9a (a histone methyltransferase inhibitor) inhibition in human gastric cell lines (AGS, SNU-216, NCI-N87, SNU-638, and MKN-74) [89].

Moreover, kaempferol has also been evaluated for its potential to target histone modifications in aggressive TNBC. Their study indicated that, as per their network pharmacology analysis, the anticancer activity of kaempferol in their model of MDA-MB-468 cells, demonstrated by DNA damage, S-phase cell cycle arrest, and suppression of cancer stemness, might be related to the inhibition of sirtuins (SIRT4 and 6), nicotine adenine dinucleotide-dependent HDACs, involved in various cell signaling pathways. They confirmed their molecular dynamics by downregulating SIRT3 and SIRT6 in kaempferol-treated MDA-MB-468 cells [113].

The development of various synthetic modulators of epigenetic modifications across multiple cancer types indicates the dire need to further evaluate further the ability of kaempferol to target various epigenetic pathways in breast cancer.

### 5.8. Effect of Kaempferol on the Tumor Microenvironment and Immune Response

Immune cells have been researched as potential targets for many anticancer therapies. The involvement of immune cells and evasion of immunosurveillance by cancer cells is prominently involved in the development and progression of cancer, where immunosurveillance or immunoediting is a process where the immune cells perform their function and reject the malignant transformation of the cells, thereby inhibiting tumor growth. Prolonged immune responses and chronic inflammation in the body are responsible for cancer development by critically modulating the protective effects of immune cells [114]. In their tumor microenvironment (TME), the cancer cells interact with various other conditions of TME, such as oxidative stress, dysregulated immune function, inflammation, and an acidic environment, resulting in cancer progression [115]. Within the TME, the immune cells, helper CD4 + T cells (Th1), and cytotoxic CD8 cells by their cytokine IFN-γ, produce significant antitumor effects, and conversely, the myeloid-derived suppressive cells (MDSC) and tumor-associated macrophages (TAM) along with their associated cytokines IL-1beta, IL-6, and TNF-alpha become involved in tumor progression [114]. However, a subpopulation of T cells, the regulatory T cells (Tregs), having a surface expression of transcription factors Foxp3 and CD25, conversely inhibits the activation of immune cells against cancer. The physiological function of Tregs is to suppress the effector immune response against the self-antigens in the body, thereby preventing the autoimmunity and inflammation associated with it. In TME, Tregs inhibit the activation of CD4 and CD8 T cells, compromising their anticancer activity and promoting cancer progression [114]. 

#### 5.8.1. Effect of Kaempferol on Tumor-Associated Macrophages (TAMs)

TME also consists of other immune cells, like macrophages and dendritic cells. TAMs are a population of the most abundant macrophages infiltrating tumor cells and have a prominent role in tumor progression. TAMs have two major populations of macrophages, M1 and M2, where M1 is called antitumor or good macrophages, while M2 is called tumor-promoting or bad macrophages [116]. TNF-α and high levels of iNOS characterize M1 macrophages, whereas M2 is associated with very high levels of cytokines, growth factors, and protease, along with high expression of Arginase 1 [117]. TAMs play a complex role in tumor development by secreting molecules and growth factors like platelet-derived growth factor (PDGF), VEGF, M-CSF, IL-10, and the chemokine C-X-C motif ligand (CXCL). The heterogeneity of the TAMs depends on the tumor type, polarization requirements in the TME, and variations in the TME. A single neoplasm can have M1 macrophage factors and other TAM phenotypes in different regions within the tumor. Within TME of solid tumors like TNBC, M1 possesses the ability to switch from the M1 subtype with antitumor activity to the M2 subtype, which suppresses the function of M1 and provides tissue repair function and limits the inherent ability of immune cells to recognize and kill the transforming cells. Additionally, another regulatory factor present in large amounts in a variety of T cells in TNBC is PD-1 and its receptor PD-L1, where PD-1 is a surface receptor on activated T cells and PD-L1 is its ligand expressed by various cells, including cancer cells. The binding of PD-1/PD-L1 limits the antitumor abilities of the immune cells, and their upregulation is associated with advanced stages of disease and lower survival rates in TNBC patients. TAMs with higher PD-1 expression have been reported to be related to immune suppression and reduced antitumor activity of the immune cells. The blockade of PD-1/PD-L1 binding using targeted therapy has been explored to revive the phagocytic activity of TAMs and thereby increase their antitumor action. TAMs have also been reported to be involved in enhancing the migration and invasion of tumor cells in TNBC. They have been shown to increase the secretion of serine proteases, MMPs, and cathepsin and are involved in tumor cell invasion of the tumor surroundings [117]. STAT3 is the prominent mediator of TAM expansion and polarization across the TME in breast cancer. Tumors release lactic acid, induce the expression of Arginase 1 (Arg1), CD206, and mannose receptor C-type 1, and activate the (ERK)/STAT3 signaling pathway, which further stimulates colony-stimulating factor 1 (CSF-1) and polarizes the TAMs. Apart from this, activating the STAT3 signaling pathway also triggers the release of IL-6, IL-4, and progranulin (a multifunctional growth factor), which are also involved in polarizing the TAMs [118]. Interestingly, breast cancer tumors have also been found to have unique TAMs in various breast cancer tissues. Breast cancer TAMs are also associated with CCL-2, CCl-5, and integrin-mediated disease progression to nearby organs such as the lungs [119]. Many compounds of natural origin have been described as inhibiting TAM infiltration by targeting CCL-2/CCR-2 signaling [120].

Many studies have explored a variety of natural compounds for their immunomodulatory properties by targeting infiltrating TAMs by modulating the (PI3K)/Akt/mTOR pathway. Alkaloids, flavonoids, and terpenoids have been used in multiple studies to target the STAT3/IL-6/Arg1 signaling pathway within TAMs, thereby inhibiting their expansion, infiltration, and immunosuppressive activities and exhibiting antitumor properties by blocking the production of pro-inflammatory molecules and signaling mediators within the TME [121]. Similarly, kaempferol has also shown anticancer activity in various cancer cell models by inhibiting STAT3 [122,123,124]. Likewise, in a study by Qian Yu et al., 2016, a kaempferol derivative called resokaempferol showed significant inhibition of lipopolysaccharide (LPS)-induced production of key inflammatory mediators including cyclooxygenase-2 (COX-2), prostaglandin E2 (PGE2), CCL2/MCP-1, nitric oxide (NO), iNOS, IL-1β, TNF-α, and IL-6 in primary murine macrophage culture. Resokaempferol also abrogated the activation of the JAK2/STAT3 signaling pathway in murine macrophages stimulated with exogenous interleukin-6 (IL-6) [125]. The immunomodulatory properties of kaempferol against TAMs by modulating multiple signaling pathways provide sufficient evidence for its ability to work against the different population macrophages in TNBC tumor TME. However, studies to analyze the exact mechanism adopted by kaempferol to inhibit the polarization of macrophages in TNBC TME need to be explored in future studies.

#### 5.8.2. Effect of Kaempferol on the Expression of CCL2

Inflammatory mediators like chemokines and cytokines guide the immune cells to the tumor sites. CCL2 is a chemokine that binds to GPCRs and plays a crucial role in regulating the recruitment of macrophages during processes such as wound healing, inflammation, and infection [126]. The higher expression of the chemokine CCL2/Monocyte chemoattractant proteins (MCP-1) and its receptor CCR2 is related to the primary breast tumor cells undergoing malignant transformation, and it was found to be expressed by the multiple cells of TME. This chemokine is upregulated in pleural effusions, serum, and interstitial fluids in breast TME. It is associated with high-grade disease states and a poor prognosis [127]. Multiple cell types in the TME—endothelial cells, stromal cells, and tumor cells—tend to produce CCL2, which recruits monocytes and TAMs to the tumor sites. The chemokine, through its CCL2-CCR2 axis, polarizes the monocytes to TAMs, resulting in tumor cell survival, and the inhibition of CCL2-CCR2 signaling blocks the recruitment of inflammatory cells to the tumor site, reducing tumor progression and metastasis [128]. A clinical study performed by Xiangzhou Chen, 2020 revealed that CCL2 secreted by TAMs in the TNBC TME activates AKT/β-catenin signaling, which is prominently involved in cell growth, survival, proliferation, and stem cell properties [129]. In a study by Zvi G. Fridlender et al., 2010, the authors analyzed the ability of CCL2-CCR2 blockade to augment the therapeutic efficacy of immunotherapy by suppressing TME-associated local immunosuppression in addition to boosting the T-cell immune response, which is the general effect of standard immunotherapy [130]. CCL2 has also been reported to induce resistance against immunotherapy in aggressive cancers like TNBC. Junyoung Choi et al., 2020 demonstrated that activation of the PI3K/AKT signaling pathway with NF-κB activation induces CCL2 secretion and PD-L1 inhibitor resistance, which is a promising immunotherapy [131].

**Figure 3:**
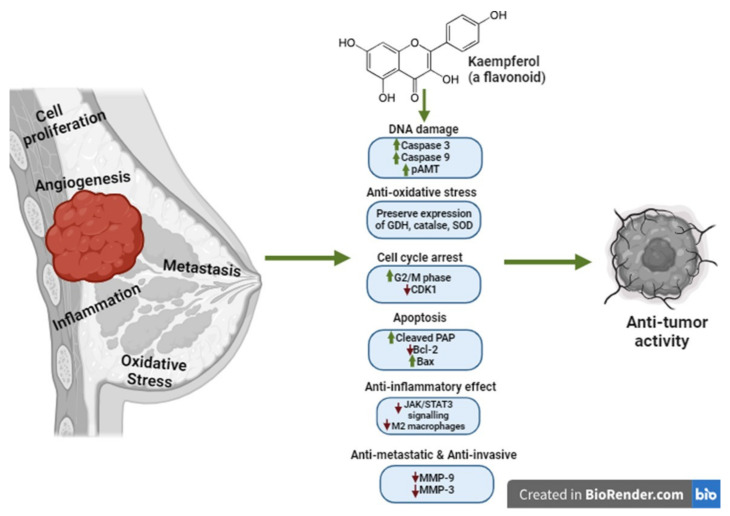
The effect of the flavonoid kaempferol on the development and progression of breast cancer. Green arrows indicate induction and red arrows indicate inhibition.

Interestingly, many compounds of natural origin have been seen to produce antiproliferative effects against genetically different TNBC cell types by inhibiting the release of CCL2 [132,133,134]. Interestingly, a computational molecular dynamic study of the analysis of the effect of flavonol-CCL2 interactions showed that flavanol compounds can attenuate the CCL2-mediated recruitment of leukocytes to the site of inflammation. In this study, kaempferol, among other flavonols (quercetin and myricetin) with multiple -OH groups in their structures, showed increased affinity towards the CCL-2 structure, demonstrating that kaempferol has the potential to reduce the CCL2-CCR2-cell surface glycosaminoglycans (GAGs)-mediated pathogenesis of inflammatory infirmities in cancer TME. More studies are still needed to explore the possibility of kaempferol modulating CCL2 in TNBC, which could reverse the drug resistance observed in the current treatments (Figure 3 and Table 1) [135].

## 6. Addressing Kaempferol’s Poor Solubility: Pharmaceutical Formulations of Kaempferol

Despite the promising anticancer activity against cancer, kaempferol still struggles with its biomedical applications due to its unstable chemical characteristics, poor water solubility and dissolution, and limited bioavailability. Quite a few efforts have been made to address these delivery-related issues of kaempferol with the development of various delivery systems, and surprisingly, they have been developed specifically for their delivery across multiple cancer types, including breast cancer. Drug delivery formulations like liposomes and nanoparticles have been extensively reported to overcome the challenges mentioned above of hydrophobic compounds like kaempferol, and they significantly improve the overall effectiveness of drugs, allowing for reduced dosages. Nanoformulations, particularly nanoparticles, have shown promising results for the biological delivery of various therapeutic agents by aiding in their bioavailability. Subsequently, the delivery of kaempferol in nanoparticles showed increased kaempferol dissolution with decreased particle size while retaining its therapeutic properties [136]. Luo et al., in 2012, developed five different types of kaempferol nanoparticle formulations and showed a significant reduction in the cell viability of A2780/CP70 and OVCAR-3 cancer cells with a lower concentration of kaempferol (25 µM) with one of their nonionic poly(ethylene oxide)-poly (propylene oxide)-poly(ethylene oxide) (PEO-PPO-PEO) nanoparticles [60]. In another study by Kazmi et al., 2021, the authors developed kaempferol-loaded nanoparticles (KFP-Np) with a quasi-emulsion solvent diffusion technique using two polymers (hydroxypropyl methylcellulose acetate succinate ((HPMC-AS) and Kollicoat MAE 30 DP) to analyze the potential of kaempferol to treat hepatocellular carcinoma (HCC) and its associated liver damage. In their in vivo model of Cadmium chloride (CdCl_2_)-induced HCC, KFP-Np demonstrated a significant decrease in the levels of elevated liver enzymes, oxidative stress markers, and antioxidant enzymes (MDA, SOD, GST, and Catalase), and also significantly downregulated pro-inflammatory cytokine expression (IL-1β, IL-6, and TNF-α) and NF-κB in comparison to HPMC-AS and free kaempferol [137]. Later, Srinivas Raghvan et al., 2015, developed kaempferol gold nanoparticles (KAuNPs) using the reactive -OH group in the catechol ring of kaempferol and showed excellent biocompatibility with biological systems. They further observed a significant increase in the cytotoxic potential of KAuNPs compared to individual kaempferols in MCF-7 cells. Also, they demonstrated the induction of apoptosis and the anti-angiogenic potential of KAuNPs [138]. Similarly, Govindaraju et al., 2019, demonstrated the cytotoxic activity of kaempferol-conjugated gold nanoclusters (K-AuNCs) in A549 lung cancer cells and showed the induction of apoptosis and inhibition of cell migration in A549 cells [139]. 

Interestingly, the more advanced version of nano-formulations has also been developed to deliver kaempferol across the blood–brain barrier (BBB). Colombo et al., 2018, prepared kaempferol-loaded nanoemulsions and targeted the delivery of their payload, the kaempferol, to the brain intra-nasally to show its anticancer activity in their in vitro and in vivo models of glioma. The formulations were prepared with chitosan (for mucoadhesion and called mucoadhesive nanoemulsion (MNE) and without chitosan, called nonemulsion (NE)) to assess the impact on nasal targeting and antitumor activity against glioma cells. Their ex vivo diffusion studies revealed significantly higher kaempferol permeation across the mucosa with MNE compared to NE. The histopathological evaluation indicated the safety of both nanoemulsions for the nasal mucosa, with no compromise with the antioxidant activity of kaempferol. MNE significantly enhanced drug delivery in the in vivo model following intranasal administration, achieving 5 and 4.5-fold higher levels compared to free kaempferol and NE, respectively [140].

Furthermore, MNE exhibited superior antitumor activity against C6 glioma cells, inducing apoptosis more than free KPF or KPF-NE. The oral delivery of kaempferol using a carrier system to increase its bioavailability has also been reported in various studies with promising results. Qian Du et al., 2019, prepared different formulations of N-trimethyl chitosan (TMC) grafted with medium- and long-chain fatty acids and observed that TMC nanoparticles grafted with decylic acids (a medium-chain fatty acid) showed enhanced cellular uptake and intestinal absorption and showed great potential for the development of such formulations of hydrophobic compounds like kaempferol [141]. Furthermore, developing more advanced formulation types, like extracellular vesicles or exosomes, for delivering synthetic and hydrophobic natural compounds has also been explored in cancer and other chronic diseases. Exosomes as a delivery system mimic the cells of their origin and provide more targeted delivery of the therapeutic agent by offering better biocompatibility and low immunogenicity and toxicity [142]. Developing smart/engineered exosomes is another upcoming technology that can deliver the desired therapeutic agents very precisely at the targeted sites, eliminating the undesired toxicity. Great options must be explored to provide a compound like kaempferol, potentially targeting the deadliest cancer type (Table 2).

## 7. Clinical Translation of Kaempferol as an Anticancer Agent

Despite much literature on the therapeutic efficacy of kaempferol against cancer and other chronic diseases, few studies address kaempferol’s potential to be evaluated and used clinically. The widely reported efficacy of kaempferol in various in vitro and in vivo studies, mainly due to its anti-inflammatory, antioxidant properties, and anticancer activity due to induction of cell cycle arrest, apoptosis, and suppression of angiogenesis, demands the dire need to replicate the results in clinical studies. A toxicological, clinical study has addressed the toxicology profile of kaempferol in clinical settings [143]. Minoru Akiyama et al., 2023, in their study, investigated the safety of high-dose kaempferol aglycone (a kaempferol derivative) in healthy adults in a randomized, double-blind, placebo-controlled study in which the participants were given either a daily 50 mg kaempferol aglycone or a placebo for 4 weeks. The authors reported no significant differences between the kaempferol and placebo groups for measurements like body size and proportions, blood pressure, blood tests, urine tests, or any adverse events. They suggested that 50 mg of kaempferol aglycone daily for four weeks is safe for healthy adults [143].

Various researchers have investigated the anti-inflammatory effects of kaempferol by using cruciferous vegetables with high kaempferol content as an intervention. In a clinical trial, healthy adults were fed diets containing different amounts of kaempferol from cauliflower, broccoli, and radish and were evaluated for various markers at 14 days. In the study, those who consumed more kaempferol (broccoli) showed significantly lower levels of the inflammatory markers IL-6 and IL-8 [144]. A study examined male smokers who consumed a broccoli diet for 10 days. This group showed decreased inflammatory markers of TNF-α and IL-6 compared to the control group [32]. Kaempferol has also shown promising anticancer activity through its anti-inflammatory activity in various in vitro and in vivo models. While all this preclinical data supports kaempferol’s role in human cancers, its clinical efficacy remains uncertain [32,35,145].

Although noticeable progress has been made to show the anticancer activity of kaempferol, resistance to standard chemotherapeutic drugs like 5-FU is still the major reason for treatment failure in human cancers like colorectal cancer. In this regard, studies have shown that combinatory therapy is a successful approach to inhibiting drug resistance. Riahi-Chebbi et al., 2019 examined the anticancer activity of kaempferol, alone or in combination with 5-FU, on human 5-FU-resistant LS174-R colorectal cells. The data indicated that kaempferol can reverse 5-FU resistance in LS174-R cells, promoting apoptosis and cell cycle arrest, hindering the production of ROS, and regulating the activation of several signaling pathways, indicating that kaempferol could be used as a possible chemotherapeutic agent to be used solely or in combination with 5-FU to reverse drug resistance [68]. In their review of kaempferol, Nejabati et al., 2022 emphasize the need for more clinical studies to unravel the therapeutic potentials of kaempferol in cancers because, although there are many preclinical studies proving kaempferol effects against different cancers, there are still numerous doubts regarding its therapeutic potency in cancer therapy [145].

To fully realize the therapeutic potential of kaempferol as an anticancer agent, further clinical trials are warranted for different human cancers, including breast cancer. Additionally, kaempferol-loaded delivery systems like nanoparticles also present a promising avenue for enhancing its bioavailability and therapeutic efficacy in cancer treatment, which can serve as another avenue to develop kaempferol as a full-blown anticancer agent.

## 8. Critical Areas for Future Research

### 8.1. Extrachromosomal Circular DNA (ecDNA) as a Target in Cancer Therapy

Extrachromosomal circular DNA (ecDNA) is a new target emerging in the scientific community as a potential genomic modifier resulting in aberrant oncogenic expressions. ecDNAs are a particular class of circular DNA that exists outside the chromosomes and is shed from the genomic DNA. It is almost 1 MB and can be non-coding and oncogene-expressing DNA [146]. They have been reported to be involved in increased copy numbers of the oncogenes, tumorigenesis, tumor heterogeneity, and drug resistance in the advanced stages of various cancer types [147]. Many proto-oncogenes in aggressive tumors have been reported to be linked to ecDNA. Additionally, it has been reported that the heterogeneity in resistant tumors is also related to ecDNA. With the evolution of recent technologies to evaluate novel mechanisms like ecDNA and its potential to modulate the occurrence and progression of cancer, it has become an important target to be studied extensively. The ecDNA presents itself as a novel target for cancer-specific therapies. It holds potential for either standalone therapeutic intervention or as a synergistic partner to augment the efficacy of established treatment modalities like radiotherapy, chemotherapy, and conventional anticancer drugs. The unique structural and functional characteristics of ecDNA in tumor development make it an attractive avenue for developing targeted drugs [148].

Furthermore, intratumorally and intertumoral variations in ecDNA levels could be exploited to design personalized treatment strategies for individual cancer patients [149,150]. A limited number of studies address the development of ecDNA as a target for various treatment modalities. Still, with the emerging information and data across multiple databases, ecDNA can be considered a novel therapeutic target for different treatment options. Similarly, using a natural compound like kaempferol, with immense potential and promising activity to modulate the activity of ecDNA in aggressive breast cancer stages, can be a therapeutic breakthrough in the treatment of breast cancer [151].

### 8.2. The Regulatory Role of MicroRNAs (miRNAs) in Breast Cancer

MicroRNAs (miRNAs) are small sets of non-coding RNA that regulate an array of molecular functions and are considered post-transcriptional gene regulators in normal cellular processes and carcinogenesis. They are widely found in the eukaryotic genome and are believed to constitute about 1 to 2% of the known eukaryotic genome. A single miRNA can modulate the gene expression of multiple genes and vice versa; more than one miRNA can coordinate multiple pathways to alter the gene expression of only one gene (oncogene in the case of carcinogenesis) and hence holds a considerable role in mediating normal eukaryotic cells and cancer cell functions. Breast cancer has a unique set of miRNAs regulating survival and cancer cell death [152]. Prominently, three miRNAs, miR-21, miR-210, and miR-221, have been seen to be considerably upregulated in TNBC and have also been associated with poor progression-free survival. At the same time, miR-10b, miR-145, miR-205, and miR-122a have been reported to be under-expressed in breast cancer cases [153].

Moreover, different miRNAs are linked to modulating different cellular functions; for example, the upregulation of miR-7-5p is related to the induction of apoptotic cell death and suppression of cell proliferation in breast cancers; over-expression of miR15a and miR-16 is linked to increased release of cytochrome c in the cytosol, activation of caspase-3 and caspase-6, and ultimately resulting in the induction of apoptosis; over-expression of miR-20a is linked to the decreased activity of the autophagy pathway; this is also related to the high copy number variations and mutations in the TNBC cells. Similarly, the downregulation of miR-23a is associated with decreased invasiveness and migration of TNBC cells. Also, the levels of miRNA regulated the chemo-sensitization of the breast cancer cells towards standard chemotherapeutic agents like carboplatin, paclitaxel, etc. [152,154]. 

Interestingly, kaempferol has been seen regulating the levels of various miRNAs, thereby affecting the survival of cancer cells, in quite a few studies. Kaempferol was reported to inhibit the cell proliferation and migration of the HeG2 liver cancer cells by downregulating the expression of miR-21, which is upregulated in advanced and aggressive breast cancer stages. They showed that by reducing the expression of miR-21, kaempferol upregulated the expression of PTEN, thereby inhibiting the signaling of the PI3K/AKT/mTOR pathway in HeG2 cells. Janet Alejandra Gutierrez-Uribe et al., 2020 reported that kaempferol-3-O-glycoside isolated from black beans showed downregulated expression of miR-31 and miR-92a along with the KRAS oncogene and increased expression of tumor suppressor genes, APC and AMPK in RKO colon cancer cells [155]. Similarly, kaempferol has been seen to upregulate the expression of miR-181a and inhibit the MAPK/ERK and PI3K pathways, resulting in inhibiting cell proliferation and inducing autophagy in SNU-216 gastric cancer cells [124]. Kaempferol has also been seen as chemo-sensitizing the resistant HCT8-R colorectal cancer cells by upregulating the expression of miR-326 and inhibiting the expression of PKM2 in glycolysis [69]. 

Although the literature shows different studies on the kaempferol effect on miRNA, none of them show the anticancer effect of kaempferol by modulating the expression of miRNAs in TNBC. The potential of kaempferol in modifying the expression of various miRNAs in different human cancer types fuels the idea of utilizing kaempferol to target miRNA-based anticancer mechanisms.

## 9. Conclusions

**Figure 4:**
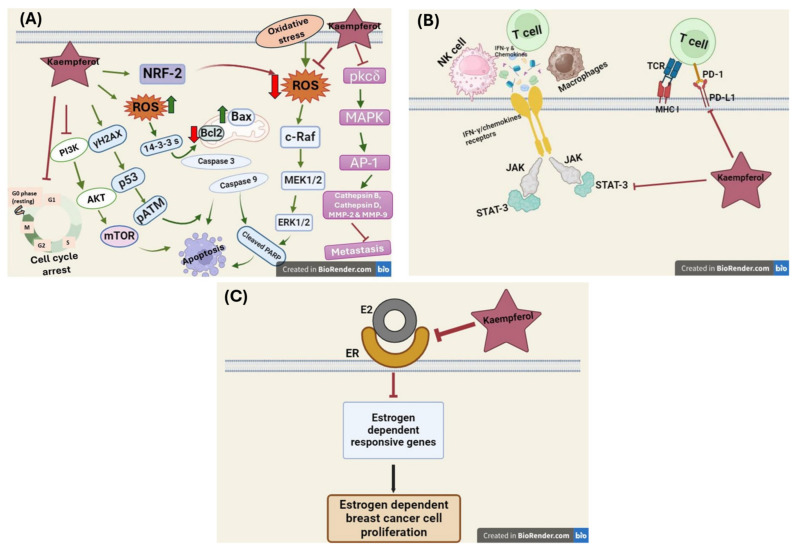
Summary of Pharmacological Effects of Kaempferol. (**A**) Oxidative stress modulation, apoptosis induction, and cell cycle arrest; (**B**) effect on inflammation and immune cells; (**C**) anti-estrogenic effect. Red arrows indicate inhibition, and green arrows indicate induction.

Breast cancer is one of the leading causes of morbidity and mortality in adult females of different age groups. Its heterogeneous nature, among other aggressive types of human cancers, makes it particularly challenging to treat, particularly at later stages of the disease. TNBC is an even more aggressive subtype of breast cancer, which further limits the availability of treatments and clinical outcomes of standardized therapies due to its differential molecular nature. Advanced research in the last few years has led us to a better understanding of the immunological and molecular heterogeneity in TNBC. It has increased the dire need to develop and explore more targeted and effective treatments with limited compromise on patients’ overall health. As a potent natural compound of enormous importance, kaempferol has been evaluated by different groups for different cancer types. Like all compounds of natural origin, kaempferol modulates multiple pathways to provide various properties, including anti-oxidative, anti-inflammatory, antiproliferative, and anti-tumorigenic properties. In human cancers, and specifically in human breast cancer, it works primarily by inhibiting DNA synthesis, modulating ROS production, inducing apoptosis, and inhibiting the angiogenic and metastatic potential of breast cancer tumors (Figure 4). Besides its potential antitumor properties, kaempferol has poor solubility and dissolution properties and limited bioavailability. This review highlights some prominent delivery systems and formulations developed to better deliver kaempferol in various cancerous and non-cancerous conditions, with increased bioavailability and targeted delivery. This review also demonstrates some of the critical mechanisms targeted with kaempferol in breast cancer, particularly in TNBC. It also emphasizes the potential of kaempferol to target various epigenetic mechanisms in different human cancers, and it fuels the idea of further exploring kaempferol in modifying epigenetics in TNBC. With the scientific advances and discovery of various novel therapeutic targets, this review highlights ways of exploring the activity of kaempferol in targeting some of the most novel targets. Overall, this article re-scrutinizes the available literature on the efficacy of kaempferol as an anti-breast cancer agent and stresses the need for further investigation to better understand and fully acknowledge the capacity of kaempferol as an antitumor agent.

## Figures and Tables

**Table 1:** The anti-breast cancer properties of kaempferol in different signaling pathways.

Study	Pathway Targeted	Outcomes
**DNA Synthesis Inhibition**		
Zava et al., 2023 [78]	DNA synthesis inhibition and cell growth	Kaempferol demonstrated estrogen agonistic activity and showed cell growth and DNA inhibition (at 10 µM concentration, the 0.25 µg DNA in T47D cells).
**Apoptosis**		
Balabhadrapathruni et al., 2000 [43]	Cell proliferation, cell cycle, and apoptosis	Kaempferol inhibits cellular proliferation by targeting the G2/M cell cycle and apoptosis in MDA-MB-468 cells.
Brusselmans et al., 2005 [102]	Fatty acid synthetase pathway	Kaempferol inhibited the fatty acid synthetase enzyme, which was overexpressed in human breast cancers, reduced cell growth, and increased apoptosis.
Kim et al., 2008 [89]	Cell viability and apoptosis in 2D and 3D cultures	Kaempferol-induced apoptosis by modulating the ERK/MEK1/ELK1 signaling pathway.
**Targeting the Estrogenic pathway**		
Kim et al., 2016 [80]	E2-mediated breast cancer cell proliferation, cell cycle, and apoptosis	Kaempferol antagonized the Triclosan stimulated cell proliferation in MCF-7 cells, upregulated the expression of cathepsin, cyclin D1, and cyclin E, and downregulated the expression of Bax and p21.
Oh et al., 2006 [74]	Estrogen-dependent and estrogen-independent pathways in breast cancer cell proliferation and malignant cell transformation	Kaempferol inhibited cell proliferation via an estrogen-dependent pathway, preventing malignant transformation of the human breast cells.
Hung et al., 2004 [79]	Cell viability	Kaempferol reduced cell viability (IC_50_: 35.0 mM and 70 mM for ER-positive and ER-negative breast cancer cells, respectively), decreased ER-alpha mRNA and protein expression, and decreased the progesterone receptor, cyclin D1, and insulin receptor expression.
**Anti-Oxidative stress**		
Zeng et al., 2020 [88]	Neutrophil extracellular traps, ROS	Kaempferol inhibited the formation of NETs, thereby reducing the formation of ROS and inhibiting metastasis in breast tumors.
Afzal et al., 2023 [16]	ROS, agonism with another potential anticancer agent: Fisetin, DNA damage, and apoptosis	Kaempferol synergized the anti-oxidative properties of fisetin and activated γ-H2AX, leading to DNA damage and apoptosis.
Wu et al., 2020 [19]	ROS, ROS-mediated cardiotoxicity, endotheliotoxicity	Kaempferol reversed the vascular toxicity and cardiotoxicity caused by doxorubicin’s adverse effects by modulating the levels of the 14-3-3 s protein, which regulates ROS levels.
** Inhibition of Metastasis and Invasion**		
Li et al., 2017 [97]	Migration	Kaempferol inhibited the activation of RhoA, Rac, and GTP-binding proteins involved in microfilament arrangement, thereby inhibiting the cancer cell migration of MDA-MB-231 cells.
Li et al., 2015 [98]	TNBC cell adhesion, motility, and migration	Kaempferol (IC_50_: 204.7 mol/L) decreased the expression of MMP-2 and MMP-9 in MDA-MB-231 cells.
**Cell Cycle Arrest**		
Choi et al., 2008 [100]	Cell cycle arrest	Kaempferol downregulated the levels of CCDK1, cyclin A, and cyclin B and induced cell cycle arrest at the G2/M phase of the cell cycle in MDA-MB-453 cells.
Varinder Kaur et al., 2018 [101]	Cell cycle arrest	Chloroform extract of *Butea monosperma* (Lam.) Taub bark rich in kaempferol produced cell cycle arrest at the G1 phase of the cell cycle in MCF-7 cells.
Zhu et al., 2019 [75]	Cell cycle arrest	Lower concentration of kaempferol is required to produce cell cycle arrest at G2/M phase in MDA-MB-231 (IC_50_: 43 µmol/L) cells than in estrogen receptor-positive BT474 breast cancer cells (IC_50_: 100 µmol/L).
Kim et al., 2016 [80]	Cell cycle arrest	Kaempferol downregulated the expression of cyclin D1 and cyclin E and upregulated the expression of p21 in triclosan-treated MCF-7 cell models.

**Table 2:** Pharmaceutical formulations and delivery agents of kaempferol.

Study	Formulation	Results
Luo et al., 2012 [60]	Prepared PEO-PPO-PEO nanoparticles, poly(lactic acid-co-glycolic acid) (PLGA) nanoparticles, PLGA polyethyleneimine (PEI), poly(amidoamine) (PAMAM) dendrimer, and glycol chitosan nanoparticles	Out of all, PEO-PPO-PEO nanoparticles of kaempferol (25 µM concentration of kaempferol) showed significant cytotoxicity towards A2780/CP70 and OVCAR-3 cancer cells in comparison to kaempferol alone.
Raghvan et al., 2015 [138]	Developed KAuNPs.	KAuNPs showed tremendous biocompatibility, demonstrated increased cytotoxic potential towards MCF-7 cells, and exhibited induction of apoptosis and anti-angiogenic activity compared to the individual compound.
Govindaraju et al., 2019 [139]	Developed kaempferol-conjugated gold nanoclusters (K-AuNCs).	K-AuNCs showed preferential nuclear localization and enhanced cytotoxicity towards A549 lung carcinoma cells compared to normal human cells.
Colombo et al., 2018 [140]	Developed kaempferol-loaded nanoemulsions, MNE (made with chitosan for mucoadhesion to achieve nasal delivery) and NE (made without chitosan), for the targeted delivery of kaempferol to the glioma cells.	Both ex vivo and in vivo studies revealed significantly higher permeation across the nasal mucosa with MNE while maintaining its antioxidant activities. They also showed higher antitumor activity against C6 glioma cells than NE and kaempferol alone.
Kazmi et al., 2021 [137]	Developed KFP-Np with HPMC-AS and Kollicoat MAE 30 DP using a quasi-emulsion solvent diffusion technique.	In their in vivo model of CdCl_2-_induced HCC, KFP-Np significantly improved liver function by lowering liver enzymes and oxidative stress markers, boosting physiological antioxidant enzymes, and reducing inflammatory markers.
